# Evaluating the SAFE Tool’s Impact on Documentation and Patient Safety During Structured Ward Rounds in General Surgery: A Retrospective Study

**DOI:** 10.7759/cureus.73823

**Published:** 2024-11-16

**Authors:** Lakshmi Malvika Atluri, Kenneth Jit Chong, Maya Zumot, Ketan Kantamaneni, Suresh Kondi, Havil Stephen Alexander Bakka, Reshmitha Kantamneni

**Affiliations:** 1 General Surgery, Royal Blackburn Hospital, Blackburn, GBR; 2 General Surgery, Royal Salford Hospital, Manchester, GBR; 3 Urology, Royal Blackburn Hospital, Blackburn, GBR; 4 Trauma and Orthopaedics, East Kent University Hospitals NHS Foundation Trust, Ashford, GBR; 5 Trauma and Orthopaedics, Queen Alexandra Hospital, Portsmouth, GBR; 6 Neurosurgery, Royal Sussex County Hospital, Brighton, GBR; 7 Medicine, Rangaraya Medical College, Kakinada, IND

**Keywords:** clinical documentation improvement, general surgery department, standardized template, ward round, ward round proforma

## Abstract

Aim

Effective documentation of critical clinical information is vital for patient safety and timely discharges. Ward rounds (WRs) are crucial for multidisciplinary assessments and care planning. Current emergency surgical WR documentation is inconsistent; therefore, this study will implement a structured WR template adapted from the Royal College of Surgeons of Edinburgh’s “Surgical Assessment for Emergencies Ward Round Tool” (SAFE) to address these shortcomings.

Methods

A retrospective review of case note entries from surgical WRs was conducted between April 1 and April 14, 2024. A total of 500 random WR entries were reviewed. Recommended standards of WR documentation were obtained from the SAFE tool. The overall documentation of 14 parameters was checked. The WR entries from the weekends have been excluded from the study. After the implementation of the template, another review of 500 case note entries was conducted between October 1 and October 14, 2024.

Results

The only consistently documented parameter is the name of the consultant (97%). Parameters such as VTE prophylaxis (5%), examination findings (18%), NBM/nutrition (20%), the patient’s current clinical status (30%), and NEWS/observations (35%) were very suboptimally documented. Management plans and discharge planning were not efficiently detailed (<30%).

All the parameters that were reviewed post-implementation of a WR template were documented, with the average being 88.14%, thus demonstrating a significantly high impact.

Conclusion

A modifiable documentation template was created to improve and standardize the General Surgery WR documentation. The implementation of a WR template has enhanced the documenting of essential elements of patient care. It enhances patient safety as well as communication and documentation, ensuring that critical issues are not overlooked during patient assessments on WRs.

## Introduction

Ward rounds (WRs) are critical in patient care, especially in general surgery, since they give the primary chance to monitor patient progress, plan therapy, and address new clinical concerns. Surgical patients may have complicated and rapidly changing demands requiring close post-operative monitoring, complication management, and interdisciplinary care coordination. Despite the importance of WRs in ensuring patient safety and fostering excellent care, many general surgery wards nationwide lack a consistent strategy for WR documentation [1]. This frequently leads to uneven procedures, with each hospital trust adhering to its protocols, potentially leading to paucity in treatment continuity and patient safety. Comprehensive documentation during WRs is essential not just for medico-legal reasons but also to address all vital elements of patient care [2]. Adequate documentation creates a clear record for future healthcare professionals, making communication easier and lowering the risk of missed or delayed treatments. According to research, unstructured or poorly documented WRs are linked to increased adverse events, fragmented communication, and a lack of accountability among the care team [2,3]. A standardized, systematic approach to WR documentation may reduce these hazards by encouraging consistency, thoroughness, and clarity in patient assessments and care planning [3]. To address this gap, the Royal College of Surgeons of Edinburgh’s Patient Safety Board and the Royal Infirmary of Edinburgh collaborated to create the SAFE tool, a documentation-based modifiable WR tool to improve the quality and consistency of WRs. The SAFE tool provides a structured template for documenting essential parts of patient care, ensuring that all pertinent clinical data is documented and reviewed methodically. The SAFE tool, which guides surgical teams through a standardized checklist, aims to improve patient safety, promote continuity of care, and foster a culture of accountability and quality improvement in general surgery wards [2,3].

The conduct of surgical WRs in the NHS varies significantly, and there is a high risk of error. Safety checklists are used in various fields other than medicine, including the aviation industry, to standardize performance and reduce human factor-related errors. These principles have been tailored to their application in health care. Checklists facilitate completing complex tasks and eliminate omissions and variations in practice, all while improving team communication, performance, and the patient experience [1]. The WHO safety checklist is an excellent example of increasing patient safety while lowering surgical mortality and morbidity. In this retrospective study, we examine the effect of establishing structured and improved documentation using the SAFE tool on patient care outcomes in a general surgical context. We investigate how this documentation tool affects WR job completion rates, continuity of care, and patient safety indicators. By exploring these outcomes, this study hopes to highlight the potential benefits of using the SAFE tool as a standardized method for WRs, which will help enhance documentation practices, patient safety, and overall surgical care quality.

## Materials and methods

Study design

This study was developed as a retrospective original research project carried out at Royal Blackburn Hospital, which is part of the East Lancashire NHS Hospital Trust. The goal was to assess the usefulness of the SAFE tool, a structured recording tool for WRs, across all general surgery wards, including the Acute Surgical Admission Ward, Colorectal Ward, and Hepato-Pancreato-Biliary (HPB) Ward. The retrospective nature of this study allowed for the examination of existing case notes, offering insights into documentation methods before and after the implementation of the SAFE tool.

Inclusion and exclusion criteria

The study included all patients admitted to the general surgery department during the study period. The selection criteria included all surgical patients, ensuring a thorough review of the WR documentation practices. The study’s goal was to capture a representative sample of documentation quality and procedures in general surgery wards by analyzing a diverse patient group from several general surgical sub-specialties. The WR entries from the weekends have been excluded from the study. Weekend entries for general surgery ward consultation were omitted from the analysis because of markedly diminished staffing numbers during the weekend period. This decision sought to improve data dependability and the quality of patient care evaluations, as insufficient staffing could adversely affect documentation and overall patient management results.

Intervention

The intervention included the use of the SAFE tool, which is a rigorous checklist designed to improve the structure and quality of WR documentation. The checklist includes crucial components such as the name of the consultant in charge of the patient’s care, detailed patient information, pertinent clinical findings, and a defined management plan. This systematic approach attempts to encourage thoroughness and accountability in documenting patient care during WRs.

Data collection

Data was collected by evaluating 500 random case note entries from all surgical wards between April 1 and April 14, 2024. After the implementation of a WR template, data was collected by reviewing 500 case note entries between October 1 and October 14, 2024. To ensure the credibility of the data collected and to reduce selection bias, two authors participated in the data extraction procedure. The data collected was indicative of overall documenting procedures throughout the wards. This approach enabled a thorough examination of documentation quality before and after the implementation of the SAFE tool.

Outcomes

The primary outcome of this study was to review an increase in documentation quality across all checklist components following the introduction of the SAFE tool. This includes evaluating the completeness and clarity of each section of the documentation as reported in the case notes. Secondary outcomes examined healthcare provider satisfaction, specifically the viewpoints of consultants and registrars on the new documentation methods. Surveys or informal feedback mechanisms were used to assess their satisfaction with the SAFE tool’s structure and usability during WRs.

Statistical analysis

The data analysis took a comparison approach, comparing the quality of documentation before and after implementation. Descriptive statistics in IBM SPSS Statistics, version 29.0 (IBM Corp., Armonk, NY), were used to summarize the collected data, with an emphasis on the number of successfully completed template items before and after the SAFE tool was implemented. Statistical tests, such as paired t-tests or chi-square tests, were used to assess the significance of the changes in documentation procedures. The purpose of this analysis was to provide insight into the SAFE tool’s effectiveness in improving the overall quality of WR paperwork and increasing healthcare provider satisfaction.

Using this rigorous methodology, the study aims to provide significant information about the influence of structured documentation tools, such as the SAFE tool, on patient care and safety in general surgery. The findings are intended to guide future practices and the use of standardized documentation methods in surgical settings.

## Results

Five hundred case notes were evaluated and included in the study between April 1 and April 14, 2024, before introducing the surgical WR template. The patient group comprised 58% men (n = 58) and 42% women (n = 42). The ward-round notes included all surgical wards, including general, colorectal, hepatopancreatic, and emergency surgical admissions. WR information was documented by junior doctors at various levels, including foundation year 1 (F1), foundation year 2 (F2), core trainee (CT), and junior clinical fellow (JCF) doctors. F1 doctors documented 50% of the instances, whereas F2 doctors accounted for 28%. JCFs and CTs accounted for 22% of the documentation (Table 1). This distribution demonstrates the junior doctors’ coordinated effort to preserve the quality of patient records throughout the WR.

**Table 1:** WR documentations WR, ward round

Documented by	Percentage
F1	50
F2	28
CT/JCF	22

**Figure 1:**
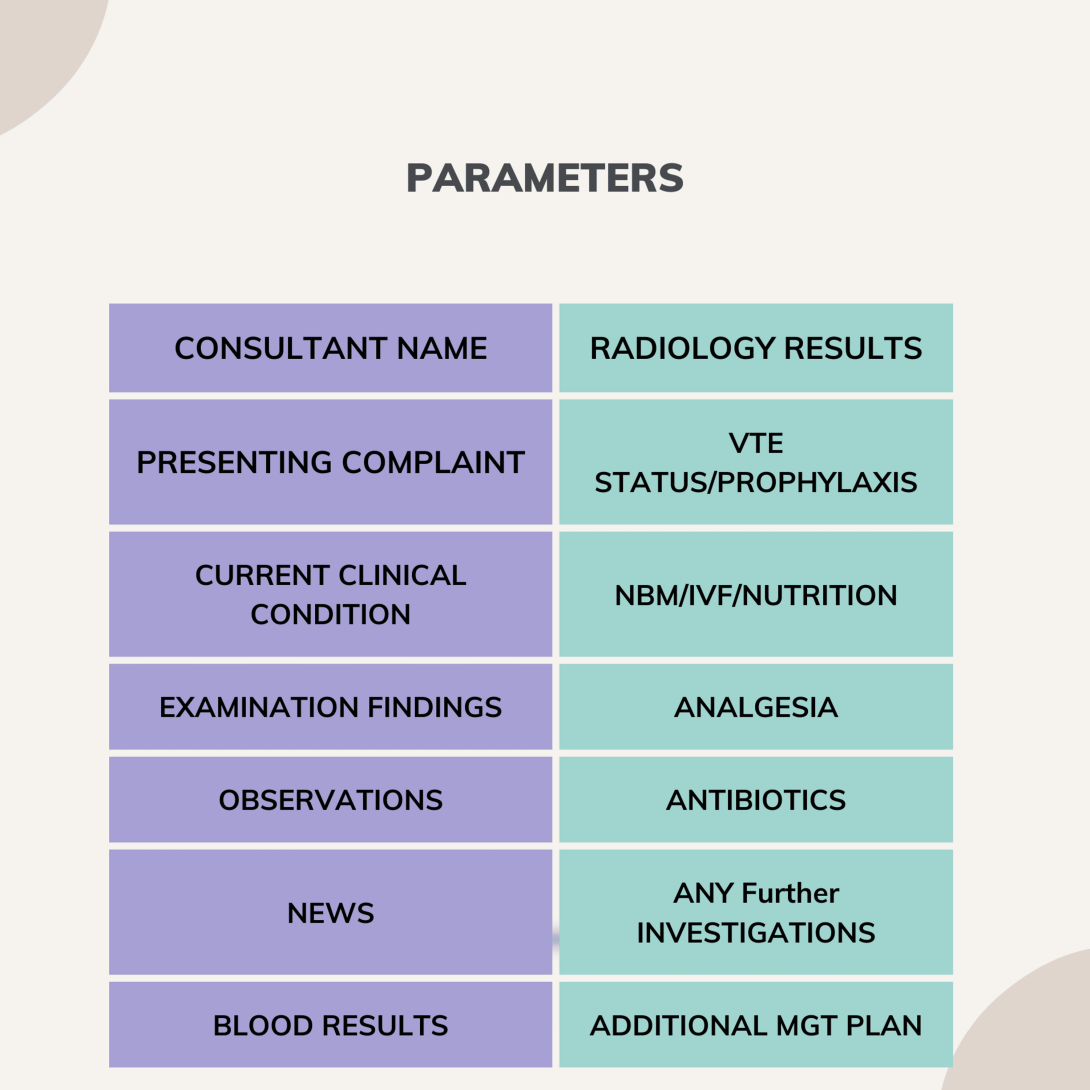
Parameters assessed on surgical WR WR, ward round

The following parameters were evaluated in this study, along with the results, before implementing the WR template (Figure 1, Table 2).

**Table 2:** Percentage of documentation of each parameter before implementation of WR template VTE, venous thromboembolism; NBM, nil by mouth; IVF, intravenous fluid; MGT, management; WR, ward round

Parameter assessed	Yes (%)	No (%)
Consultant name	97	3
Presenting complaint	41.7	58.3
Current clinical condition	30	70
Examination findings	18	82
Observations	35	65
News	35	65
Blood results	16.7	83.3
Radiology results	30	70
VTE prophylaxis/status	5	95
NBM/IVF/nutrition	20	80
Analgesia	6.7	93.3
Antibiotics	23	77
Any further investigations	23.3	76.7
Additional MGT plan	95	5

Consultant identification

Overall, 97% of notes included documentation for the consultant in charge of the patient’s care. This displays diversity in clinical oversight, which is required to understand patient care thoroughly. Documenting a consultant’s name is relevant, as escalating in a deteriorating patient would be quick. The patient’s presenting problems were only noted in 41.7% of notes. The most prevalent presenting problems were abdominal pain, nausea, and vomiting. Documenting the presenting complaints is crucial to monitor improvement in clinical condition and relief of symptoms. Current history was documented in only 30% of notes. This metric is crucial because it detects patient status changes and helps guide management decisions during ward visits. Examination findings were recorded in 18% of instances. The depth of this documentation varied, with some entries providing lengthy assessments and others being minimal. Comprehensive examination records are essential for continued patient evaluation and care. Patient observations, including vital signs, were documented in 35% of the notes. This is an essential component in monitoring patient stability and recognizing problems. The National Early Warning Score (NEWS) was calculated and documented for 35% of instances. The NEWS helps detect patients at risk of deterioration, and its constant application is critical in surgical wards. Blood results were documented in 16.7% of notes. These findings are critical for informing treatment decisions, particularly in the postoperative situation.

Radiology results were recorded in 30% of patient notes. These findings substantially impact management tactics and surgical planning, emphasizing the importance of careful documentation.

Prophylaxis for venous thromboembolism (VTE)

5% of notes had information on this topic. This documentation is essential given the significance of preventing thromboembolic events in surgical patients. Documenting VTE status in WR records is vital because it supports the proactive assessment and mitigation of thrombotic risks, an essential component of postoperative treatment. Accurate VTE documentation allows doctors to accurately stratify patient risk, resulting in timely preventative measures that reduce morbidity and mortality associated with thromboembolic events.

Fluid balance

Records were kept in 20% of cases, providing insight into patient input/output, nil by mouth (NBM) status, intravenous fluid (IVF) prescription, and directing subsequent care. Details on analgesia were reported in a mere 6.7% of the notes. Effective pain management is a critical component of postoperative care. Antibiotic administration was documented in 23% of instances. This is especially crucial for treating abdominal sepsis. Clear documentation of dose, route, and duration is of paramount importance.

Additional investigations required

23.3% of notes indicated further investigations were necessary. Identifying extra diagnostic needs is critical for providing thorough patient care. Additional management (MGT) plans, such as discussions with other specialties or teams, were provided in 95% of the notes. This strategy is critical for guiding future care and ensuring continuity in patient management.

**Figure 2:**
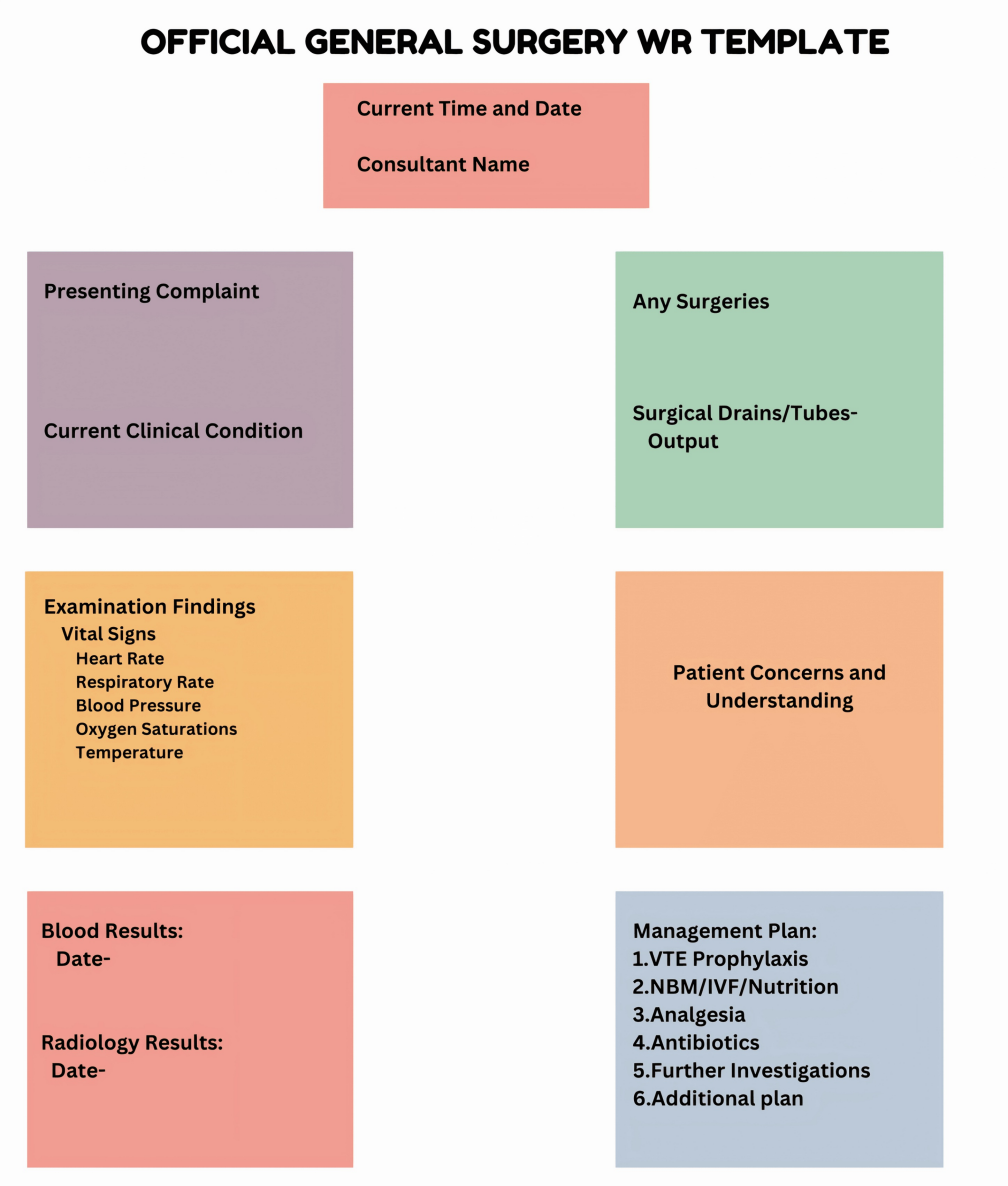
WR template based on the SAFE tool WR, ward round

Following the use of the surgical template modified from the SAFE tool (Figure 2), there was a significant improvement in documentation quality across all 14 parameters.

This modification permitted an organized process, enabling the thorough capture of crucial data. The comprehensive documentation, which had previously been inconsistent, garnered an average compliance percentage of 88.14% (P < 0.005), indicating a significant improvement in thoroughness and consistency. A thorough comparison between differences in documentation pre- and post-WR template is depicted in Table 3. The template significantly improved patient care quality, increasing transparency and facilitating postoperative assessments and outcomes tracking.

**Table 3:** Difference in documentation pre- and post-template VTE, venous thromboembolism; NBM, nil by mouth; IVF, intravenous fluid; MGT, management

Parameter assessed	Yes (%) pre-template	Yes (%) post-template
Consultant name	97	100
Presenting complaint	41.7	80
Current clinical condition	30	78
Examination findings	18	88
Observations	35	100
News	35	100
Blood results	16.7	90
Radiology results	30	92
VTE	5	80
NBM/IVF	20	76
Analgesia	6.7	75
Antibiotics	23	85
Any further investigations	23.3	90
Additional MGT plan	95	100

## Discussion

Implementing the customized SAFE tool template was intended to solve the documentation issues by ensuring that all critical clinical parameters were consistently examined and reported. Documenting the consultant’s name increased marginally from 97% to 100%, indicating universal compliance. However, significant improvements were observed in parameters with lower initial compliance. The documentation of presenting problems increased from 41.7% to 80%, while the documentation of the patient’s current history improved from 30% to 78%. Examination findings increased significantly from 18% to 88%, demonstrating how the template contributed to a more thorough clinical examination. Other crucial metrics showed significant improvements. The recording of patient observations (heart rate, blood pressure, respiration rate, and oxygen saturation) increased from 35% to 100%. In comparison, NEWS score documentation climbed from 35% to 100%, highlighting the template’s importance in improving patient safety through consistent, complete observations. Similarly, blood result documentation improved considerably from 16.7% to 90%, and radiology results (including ultrasound, CT, and X-ray findings) increased from 30% to 92%, ensuring that essential diagnostic information is easily accessible to influence clinical decisions.

The template significantly influenced interventions, including VTE prophylaxis, which went from 5% to 80%, and fluid balance, which climbed from 20% to 76%. Furthermore, medication-related characteristics, such as analgesia and antibiotics, improved from 6.7% to 75% and 23% to 85%, lowering the likelihood of prescription errors. Documentation of additional investigations necessary increased from 23.3% to 90%, while documentation of additional management plans improved from 95% to 100%, indicating that complete management plans were accurately recorded.

WRs are essential for multidisciplinary teamwork in hospitals, as they help monitor patient progress, plan and coordinate treatment, and make educated management decisions [2]. For these rounds to be effective, proper documentation is required for medico-legal reasons and to ensure patient safety and effective communication across the healthcare team. The information collected during WRs directly impacts the quality of the decisions that are made. It is critical to ensure continuity of care, especially given the fluctuation of staffing and shift patterns. Good documentation is the cornerstone for effective patient care, as it bridges communication gaps and ensures consistency, particularly in settings with frequent handovers and shifting younger personnel.

During surgical WRs, the junior team member, often an FY1 or FY2 doctor, CT, or JCF, oversees documentation. The clinician leading the WR, typically a consultant or surgical registrar, frequently stops or performs numerous jobs simultaneously, making essential information easy to ignore or record incorrectly. Furthermore, even after assessments are completed, accurately documenting these observations might be difficult, increasing the potential for misinterpretation [3]. This variation emphasizes the significance of an organized approach to documentation, which a WR template can offer [4]. Initially developed as part of the Royal College of Surgeons of Edinburgh’s Surgical WR Toolkit, the SAFE tool encourages systematic, checklist-based recording that can be linked to electronic patient records, which is in line with our hospital’s digital infrastructure. By including prompts for critical clinical elements, such as VTE prophylaxis, fluid management, antibiotic prescriptions, and pain control, the template helps junior clinicians conduct thorough assessments and capture a complete picture of each patient’s status and management plan. To aid the template’s implementation, education sessions were held to emphasize its importance and ensure junior doctors understood its role in improving patient safety. Following its installation, we saw significant improvements in documenting crucial clinical indicators.

Influence on communication and multidisciplinary collaboration

A standardized template has also improved communication between patients and the nursing team. With a checklist in place, junior doctors are more consistent in informing patients and conveying care plans, which improves patients’ understanding of their treatment and builds trust. The template’s standardization allows for accessible communication between medical and nursing professionals, lowering the possibility of misunderstandings and missing actions. In surgical settings, where patient status can change quickly, consistency is critical for permitting speedy responses to clinical changes and assuring alignment among all team members on the care plan [5].

We categorized adverse events into four domains: (1) prescription errors, encompassing incorrect prescriptions of antibiotics, analgesics, or fluids; (2) missed procedural skills, such as cannula and catheter insertions; (3) patient communication, where patients reported inadequate information or involvement in decision-making; and (4) interprofessional communication within the multidisciplinary team, which led to missed or duplicated tasks due to coordination lapses. Following the implementation of the checklist, these errors markedly decreased, as evidenced by the data. Table 4 highlights the significant pre- and post-implementation reductions in each category, underscoring the checklist’s impact on improving clinical accuracy and cohesion.

**Table 4:** Comparison of errors pre- and post-checklist

S. no.	Reported events	Pre-checklist (n)	Post-checklist (n)
1	Prescription errors	14	1
2	Skills	12	2
3	Discussion within team	14	0
4	Communication with patients	10	2

The template improved care continuity by emphasizing the significance of clear, detailed documentation. Keeping high-quality documentation is critical to preventing care failures because the information documented during one ward visit immediately influences the decisions made in subsequent rounds. In our situation, where junior doctors frequently rotate, the template provides a dependable foundation that reduces variances in documentation procedures among professionals of varying levels of expertise.

Educational advantages and skill development for junior doctors

The checklist-based template also provides educational benefits to junior doctors. It is a teaching tool that promotes a methodical approach to patient assessment, encouraging junior personnel to follow best practices in recording. The organized prompts help junior practitioners capture all important information, improving their comprehension of the clinical decision-making process. This expertise is invaluable as junior doctors advance to more independent responsibilities, providing them with the documentation skills necessary for safe and successful patient care.

Challenges and considerations for template implementation

While the template’s merits are obvious, the initial implementation was not without obstacles. As with any new tool, several clinicians expressed concern about the perceived increase in administrative effort, especially in high-pressure settings where time is restricted. However, regular education sessions, senior staff backing, and observed improvements in documentation quality have progressively led to universal adoption. Continuous feedback from physicians was also critical in developing the template to better correspond with the workflow of daily rounds, ensuring that it was user-friendly without sacrificing comprehensiveness. Regular audits are planned to evaluate template adherence and examine its long-term influence on patient outcomes. We intend to quantify the checklist’s impact on patient safety and care quality by monitoring factors such as the frequency of VTE prophylaxis omissions, prescription errors, and missing crucial information documentation. Collecting user input will also help identify any changes needed to improve the template’s usability, ensuring it effectively supports clinical workflows.

**Figure 3:**
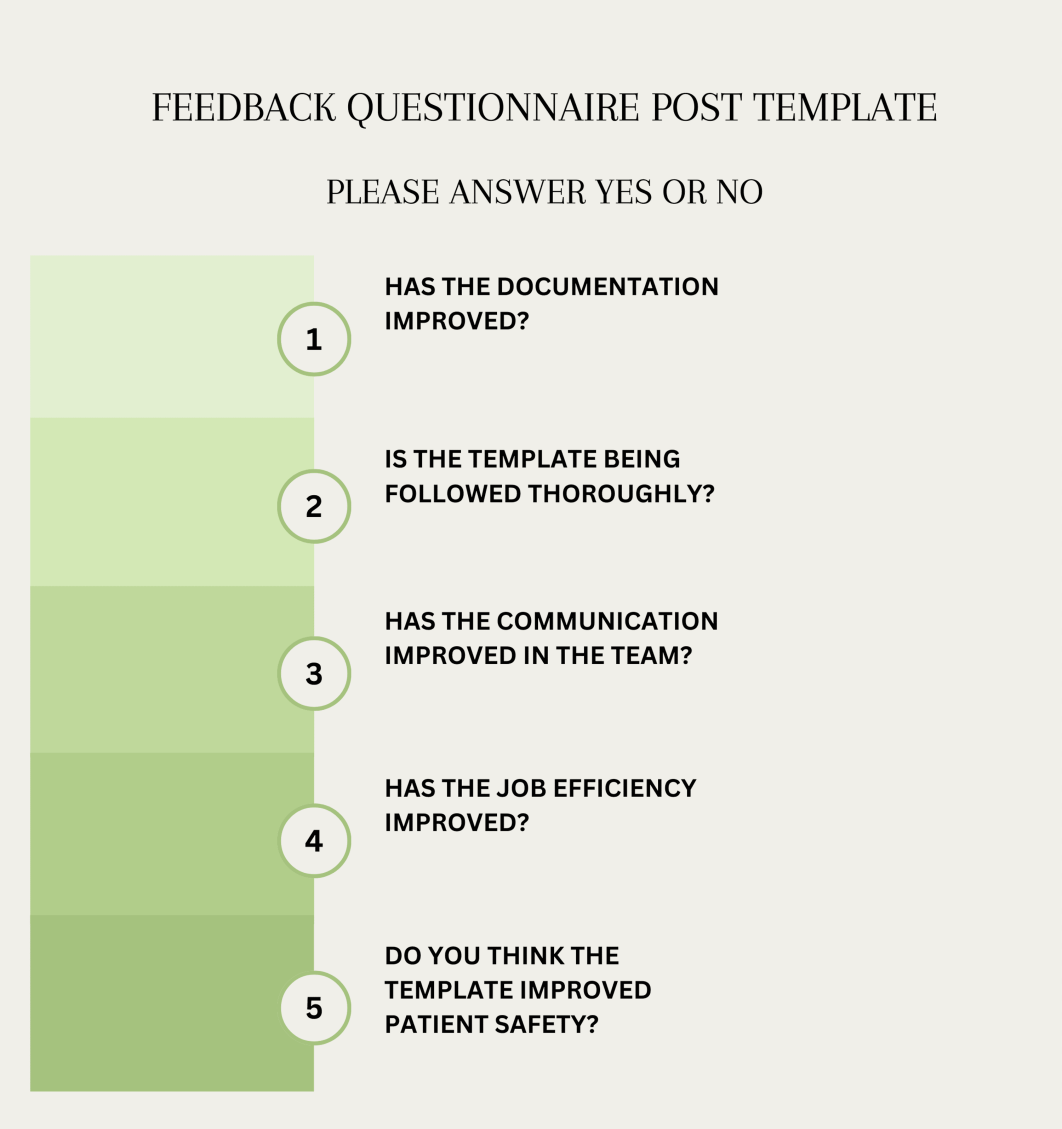
Feedback questionnaire

Seniors such as registrars and consultants claimed great satisfaction after using the customizable template, according to surveys performed with a questionnaire comprising yes/no questions where yes indicates a positive reply, implying an improvement in documentation and patient safety. The overall percentage of yes was 90% (Figure 3). This indicated that the template efficiently reduced documentation, met senior doctors’ expectations, and improved record accuracy, suggesting a positive reception and endorsement from experienced surgical team members.

Future directions and broader implications

As hospitals continue to convert to electronic health records (EHRs), including structured documentation templates in digital platforms is a viable route for improving service quality [6]. Embedding templates such as the customized SAFE tool into EHRs can help to streamline documentation procedures, decrease redundancy, and enable real-time data entry, ultimately improving the efficiency and quality of patient records.

The study has certain drawbacks. For starters, its retrospective design may bring selection and recall biases, which could have an impact on data reliability. Furthermore, the findings are based on a single center, which may limit their generalizability to other healthcare settings. Variability in staff tool adherence, as well as differences in documentation methods, may have an impact on outcomes. The short-term evaluation period may not accurately reflect the long-term impact on documentation quality. Finally, relying on subjective input to assess perceived changes may ignore quantitative measurements, emphasizing the need for more objective, longitudinal investigations.

## Conclusions

Using a structured WR template developed from the SAFE tool has been effective in addressing long-standing difficulties with inconsistent and inadequate documentation in emergency surgical wards. By providing a standardized structure for recording patient assessments and management plans, the template improves care continuity, eliminates errors, and promotes better communication among multidisciplinary teams. As healthcare settings prioritize patient safety and continuity of care, structured documentation tools such as the SAFE-inspired template can help achieve these objectives, resulting in a safer and more reliable healthcare system.
